# Is there an affective neuroscience of spirituality? The development and validation of the OCEANic feelings scale

**DOI:** 10.3389/fnhum.2024.1329226

**Published:** 2024-01-17

**Authors:** Beate Schmautz, Jürgen Fuchshuber, Deborah Andres, Theresa Prandstätter, Lisa Roithmeier, Anton Freund, Andreas Schwerdtfeger, Human-Friedrich Unterrainer

**Affiliations:** ^1^Institute of Psychology, University of Graz, Graz, Austria; ^2^Center for Integrative Addiction Research (CIAR), Grüner Kreis Society, Vienna, Austria; ^3^Department of Psychoanalysis and Psychotherapy, Medical University Vienna, Vienna, Austria; ^4^Comprehensive Center for Clinical Neurosciences and Mental Health, Medical University Vienna, Vienna, Austria; ^5^Faculty of Psychology, University of Vienna, Vienna, Austria; ^6^Department of Psychiatry and Psychotherapeutic Medicine, Medical University Graz, Graz, Austria; ^7^Department of Religious Studies, University of Vienna, Vienna, Austria; ^8^Faculty of Psychotherapy Science, Sigmund Freud University Vienna, Vienna, Austria

**Keywords:** oceanic feeling, spirituality, affective neuroscience, primary emotions, test development

## Abstract

**Background:**

Oceanic feelings represent a phenomenological structure of affective sensations that characteristically involve feelings of self-dissolution and feelings of unity and transcendence. This study presents the preliminary version of a self-report instrument to measure individual dispositions toward oceanic feelings in order to enable further research within the concept of primary emotions postulated by Jaak Panksepp.

**Methods:**

A first version of the questionnaire was applied to a total sample of 926 German-speaking adults of the general population. After performing item analysis and principal component analysis (PCA) in a first study (*N* = 300), the questionnaire was shortened. In a second study (*N* = 626), confirmatory factor analysis (CFA) was conducted and emerged scales were related to the already established instruments for the assessment of primary emotions (BANPS-GL) and Big Five personality traits (BFI-44).

**Results:**

The OCEANic scale exhibited reliabilities ranging from Cronbach’s α = 0.82 (positive) to α = 0.88 (negative) and plausible correlations with behavioral traits related to the seven affective neurobiological systems (ANGER, FEAR, CARE, SEEK, PLAY, SADNESS, and LUST) as well as with personality factors measured by the Big Five Inventory. For CFA, a bifactorial model with an overall factor demonstrated good fit: RMSEA = 0.00 (90% CI:0.00, 0.03); TLI = 1.00; CFI = 1.00; NFI = 0.99.

**Discussion:**

The OCEANic scale enables the operationalization of oceanic feelings comprising two subscales and one total scale. The results indicate good reliability and acceptable factorial validity. Establishment and further validation of the OCEANic scale within future research will be needed to fully understand the role of oceanic feelings within the human affective life, especially the personality trait of spirituality.

## Introduction

*“Some are Born to sweet delight – some are born to Endless Night”* William Blake – The Auguries of Innocence; excerpt

In 1927, Romain Rolland, a French dramatist, wrote a letter to Sigmund Freud in which he requested him to analyze a feeling. He described it as a “spontaneous religious sentiment or, more exactly, a religious feeling which is the simple and direct fact of the feeling of the eternal (which can very well not be eternal, but simply without perceptible limits, and like oceanic)” (Parsons, 1999; Abella et al., 2011, p.173). Until today, there is no clear definition of this concept. Some researchers consider the oceanic feeling to be a form of spirituality, while others – e.g., Freud – analyzed it in terms of primary narcissism meaning a state of primitive fusion with the primary object (Saarinen, 2014).

Oceanic feelings are usually classified as mystical with reference to Ramakrishna. Therefore, Saarinen (2014) suggests two distinct forms of oceanic feelings: “(1) transient episodes that consist in a feeling of dissolution of the psychological and sensory boundaries of the self, and (2) as a relatively permanent feeling of unity, embracement, immanence, and openness that does not involve occurrent experiences of boundary dissolution.” (p. 1). In the subsequent discourse, we aim to elucidate the emergence of oceanic feelings from a developmental psychology perspective and underscore the significance of considering both facets, the positive as well as the negative. Building upon Freuds ideas on early stages of psychological development, Melanie Klein introduced the concept of an early developmental phase termed the paranoid-schizoid position, which in turn is linked to the so called “primary-envy,” characterized by dreadful phantasies of persecutory objects (Klein, 1996). In many ways Klein’s formulations represent a negative counterpart to Rolland’s blissful oceanic feelings, in which Freud saw memory traces of early experiences of oneness between mother and child. Both components represent distinct facets of early childhood development that may manifest as psychopathologies later in life. Clinically, the entanglement between positively and negatively valanced states of dissolution can often be observed in the case of schizophrenia and related psychotic disorders, which frequently entail delusions of grandiosity and all-connectedness on the one hand and intense feelings of dread, fragmentation and paranoid delusions on the other hand (Andreasen et al., 1994; Unterrainer and Lewis, 2014).

In the last decades the psychoanalytic study of emotions gained new traction via the interdisciplinary field of Affective Neuroscience (AN) which investigates neuronal mechanisms of emotion, personality and mood (Unterrainer et al., 2017). Based on his studies on mammalian brains, Jaak Panksepp identified areas in the subcortical-limbic circuits of the brain to be the seat of seven primal emotion systems (Panksepp, 2004; Fuchshuber et al., 2019, 2022), which he derived from electrical stimulation of specific neuronal networks, namely SEEKING/foraging, PLAY/joy, CARE/nurturance, LUST/sexuality; PANIC/separation, RAGE/anger, and FEAR/anxiety. Within contemporary psychoanalytic discourse these affective systems are currently discussed regarding reconceptualization of classical drive theory (Fuchshuber and Unterrainer, 2020; Solms, 2021; Kernberg, 2022).

The Affective Neuroscience Personality Scales (ANPS; Davis et al., 2003) is the corresponding tool to measure behavioral traits related to the primary emotion systems. Even though spirituality is not considered a primary emotion, Panksepp recognized its relevance in human affairs, particularly in the context of addiction treatment initiatives due to its potential impact in individuals’ well-being and recovery processes as highlighted by Brienza et al. (2023). As a result, he incorporated a subscale into the ANPS to assess various aspects of spirituality, such as the importance of spiritual values or the frequency of engaging in spiritual practices. The ANPS was designed to explore the neurobiological underpinnings for the Five Factor model of personality (Davis and Panksepp, 2011) and was inspired by Cloninger’s biologically based personality theory (Cloninger, 1994).

In contrast to the dimensions of primary emotions, the spirituality scale was not linked to a specific neuronal substrate (see e.g., Seeman et al., 2003 for further discussion). However, due to its significance in investigating the meaning of oceanic feelings, we included this scale in our study.

Over the past few years, research and application of psychedelics within the clinical field (re-)gained popularity. A resting-state fMRI analysis investigated the effects of psilocybin on the connectivity between different brain networks. The results showed that psilocybin increased the connectivity between the default mode network (DMN) and the task-positive network (TPN). The researchers propose that DMN and TPN are related to internally and externally focused states, whereas these findings could explain similarities between early psychosis and the psychedelic state (Carhart-Harris et al., 2013). Regarding mechanisms, Carhart-Harris et al. (2018) propose that the psychedelic induced state of plasticity may facilitate the revision of cognitive biases when combined with psychological support in the treatment of treatment-resistant depression.

A systematic review of 12 studies showed a significant correlation between mystical experience and improved clinical outcome in nine out of twelve studies (Ko et al., 2022). Several of those studies showed a significant positive mediating effect of mystical experience on symptom reduction in patients with substance use disorder or depressive disorders. On the other hand, psychedelic experiences lead to acute anxiety in some patients that negatively influenced the therapeutic outcome of psilocybin (Roseman et al., 2018) and ketamine intake (Aust et al., 2019). In conclusion, intake of certain substances can evict both, oceanic bliss as well as intense psychological terror with both states resonating with experiences described in spiritual literature (Cross, 2007) and psychedelic research (Strassman, 1984). As this is a systematic review, mystical experiences were not measured using the same questionnaires. Generally, mystical experiences were characterized by features such as oceanic boundlessness, ego dissolution, and universal interconnectedness (Ko et al., 2022). Due to their significant similarity to our concept, this suggests the clinical relevance of oceanic feelings and the necessity for a unified questionnaire.

This leads to the question: How might these feelings – pleasurable or unpleasurable – be operationalized on a psychometric level and is there a biologically anchored disposition toward the experience of oceanic feelings like the affective traits described by Panksepp (Hiebler-Ragger et al., 2018)?

The discovery of the endogenous tryptamines like N,N-Dimethyltryptamine (N,N-DMT) and 5-methoxy-N,N-dimethyltryptamine (5-MEO-DMT) provide valuable information in this regard (Barker, 2022): Several studies linked DMT both to spiritual experiences as well as temporary psychotic symptoms in healthy participants, but also to schizophrenia (Grammenos and Barker, 2015; Dos Santos et al., 2017). On an anatomical level, a recent large-scale lesion study, suggested the periaqueductal gray (PAG) as the central node regarding the neural circuit for spirituality (Ferguson et al., 2022). The PAG plays a role in various neurobiological functions, such as regulating pain, anxiety, and reproductive behavior. The structure may be involved in maintaining a balance or transitioning information that is relevant to survival importance (Linnman et al., 2012). Furthermore, Panksepp highlighted the role of this brain area in his theory of primary emotion systems (Panksepp, 2004). For this study, we consider oceanic feelings as a potential archaic affective foundation of spirituality (Alcaro et al., 2017; Hiebler-Ragger et al., 2018). To our knowledge, currently there is no adequate measurement tool regarding the individual disposition toward oceanic feelings, at least from the perspective of AN (see Studerus et al., 2010 for further discussion).

Hence, the aim of this study is to develop and psychometrically evaluate a measurement tool for the assessment of the disposition toward oceanic feelings. For this aim, reliability and validity of this new concept will be examined. The OCEANic scale might represent the preliminary stage of spirituality, which we consider to be on the same level as the Big Five personality factors (see especially Alcaro and Panksepp, 2011 for further discussion). Therefore, this scale is expected to be positively correlated with the spirituality scale in accordance with the findings of the “Big Six” of personality (Piedmont, 1999), where the “Big Five” of personality were enhanced by means of a “Spiritual Transcendence” factor. The writing style “OCEAN” refers to the notation of primary emotions, which are commonly written in uppercase letters.

## Materials and methods

### Item generation

We developed a pool of 22 questions based on a comprehensive literature review in the field of spiritual feelings and psychedelic or psychotic experiences (see e.g., Hunt, 2007). According to “The Oceanic Feeling: A Case Study in Existential Feeling” by Jussi Saarinen, oceanic feelings describe a profound, transcendent experience that fills an individual with a sense of connection to something greater or universal. It is characterized by a feeling of boundlessness, ego-dissolution and universal interconnectedness. Boundlessness and ego-dissolution are linked to negative affects in form of mystical experiences like overwhelming dread and horror of annihilation as well (Saarinen, 2014) (see Appendix). During generation of questions, particular emphasis was laid on comprehensibility and shortness as well as avoidance of double negotiations and ambiguity (Bühner, 2011). The response format was set to a five-point Likert scale ranging from 1 to 5 (“1 = strongly disagree,” “2 = disagree,” “3 = neither agree nor disagree,” “4 = agree,” “5 = strongly agree”).

### Sample and procedure

Participants were recruited through advertising on social networks, including public forums and announcements at the University of Graz, Austria. After declaring informed consent, demographic data (age, sex, nationality, highest education, religious confession, occupation, and psychiatric history) was collected. Accordingly, a variety of standardized questionnaires was given in German language: The Brief-Affective Neuroscience Personality Scales (BANPS-GL), the Big Five Inventory (BFI-44), the spirituality scale of the ANPS (Reuter et al., 2017) and additionally, the 22 Items that were constructed in order to conduct the OCEANic feelings scale. Data of the first 300 participants (sample A) and of further participants (sample B) was used for exploration and validation phase, respectively. Data was acquired via online-survey platform LimeSurvey©. Participants were recruited via a student mailing list, public forum announcements and social media advertising that included access to LimeSurvey© via a link or QR code. In order to ensure optimal display of all questionnaires, they were asked to complete the survey on their laptop and be in a quiet environment. They had the option of temporarily interrupting the study and resuming it at a later date. The inclusion criteria were fluency in German, completion of all questionnaires and age over 18 years. The participants always remained anonymous.

Our study was carried out in accordance with the Declaration of Helsinki. Ethical approval was granted by the Ethics Committee of the University of Graz, Austria (No. 110). Participants were recruited between July 2022 and December 2022.

### Methods and analysis strategy

Metric parameters were descriptively summarized using means and standard deviations. Categorial parameters were given as absolute and relative frequencies. Calculations were performed using IBM SPSS Statistics (Version 27, 2020, International Business Machines Corporation, Armonk, NY, USA) and RStudio 2023.06.0 + 421. Principal component analysis (PCA) was conducted in order to define the underlying factor structure of our measure. In a next step, the estimation of the confirmatory factor analysis (CFA) was implemented with the R package Lavaan. Goodness-of-fit and regression weights were assessed via the robust weighted least squares (WLSMV) estimator. For further investigation regarding the construct of our measurement for oceanic feeling, we applied a bifactor model and a correlated model. We chose the bifactorial model since we are interested in whether there is a significant overlap between the items of the positive and the negative components of oceanic feelings (e.g., self-dissolution), which would justify a common factor.

Sample A (first 300 participants) was used for PCA, while sample B was used for CFA and correlation analysis. In accordance with Kline (2023), the following fit-indices were considered as markers for an acceptable model fit: (a) The comparative fit index (CFI) >0.90; (b) Tucker Lewis-index (TLI) relative fit index >0.90; (c) The square root error of approximation (RMSEA) <0.08 and the upper bound of its 90% confidence interval <1. The alpha-level was set to 0.01. Internal consistencies were calculated to measure reliability using Cronbach’s α. To facilitate subsequent replications of the study, Spearman’s correlations were performed between the mean scores of the OCEANic scale and the seven primary emotions including spirituality, as well as the Big-Five personality scale. Additionally, in order to gather further information about the overall OCEANic factor, factor scores based on the bifactorial model were calculated and included in the correlation analysis as well. Confidence Interval was set to 95% and a *p*-value of <0.05 was considered significant.

### Item reduction

We tested the initial item pool on the sample used for principal component analysis (*N* = 300). Based on the recent development of the Pleasure Scale and its integration into the BANPS in the form of BANPS-GL, we targeted a total number of 12 item for our final OCEANic scale (six items for each of the two subscales). The trimming steps were carried out iteratively by examination of item-total correlation aiming to achieve Cronbach’s α > 0.80 for the short version, as well as considerations regarding construct validity.

### Psychometric assessment

The newly adapted German version of the *Brief Affective Neuroscience Personality Scales including a LUST-scale (BANPS-GL)* was used to measure behavioral traits related to the seven affective neurobiological systems (ANGER, FEAR, CARE, SEEK, PLAY, SADNESS, and LUST) (Fuchshuber et al., 2023). This test consists of 38 items, rated on a five-point Likert scale, ranging from “strongly disagree” to “strongly agree.” Based on literature, the BANPS-GL is reliable and has acceptable to good internal consistencies ranging from Cronbach’s α = 0.69 (CARE) to α = 0.85 (SADNESS) (Fuchshuber et al., 2023).

The *Big Five Inventory (BFI)* (John et al., 1991) is a 44-item self-report inventory to measure personality according to the Big-Five-Factor-Model, namely Openness to experience, Conscientiousness, Extraversion, Agreeableness and Neuroticism as well as an additional scale for spirituality. The item response scale ranges from “strongly disagree” to “agree very well.” The German version (Danner et al., 2016) used in this study, achieves acceptable to good internal consistencies across all five personality dimensions (Cronbach’s α:0.71−0.85) (Lang et al., 2001).

As previously stated, 22 newly constructed items were given to measure positive aspects and negative aspects of oceanic feelings, respectively. The item response scale ranges from “strongly disagree” to “strongly agree.”

## Results

### Sample characteristics

In total, 928 participants completed the questionnaires, while another 638 discontinued their participation prematurely. Two persons were excluded because of missing informed consent. Mean age of sample A (*N* = 300; female: 72.7%) and B (*N* = 626; female: 68.7%) was 25 (SD ± 8) and 29 (SD ± 10.1) years. Concerning sexuality, 221 (A: 73.7%) and 475 participants (B: 75.9%) were heterosexually and 43 (A: 14.3) and 78 (B: 12.5%) bisexually oriented. Regarding relationship status, most probands were single (A: *n* = 162; 54%; B: *n* = 288, 46.0%), followed by those living in a relationship (A: *n* = 114, 38%; B: *n* = 222, 35.5%). The majority of both groups had Austrian, German or Swiss nationality (A: *n* = 274, 91.3%; B: *n* = 581; 92.8%). Most subjects’ highest educational qualification was a qualification for higher education (A: *n* = 173, 57.7%; B: *n* = 242; 38.7%). Most of the persons were currently in school, training or college (A: 243, 81%; B: 359, 57.3%), whereas 574 were employed (A: 161, 53.6%; B: 413, 66.0%). A total of 28 subjects in group A (9.3%) stated to be diagnosed with psychiatric disorders, versus to 102 (16.3%) in group B (see Table 1).

**TABLE 1:** Sample characteristics (exploration and validation phase).

Sample	Exploration phase	Validation phase
Overall	*N* = 300	*N* = 626
Gender	*N* = 218 Female (72,7%)	*N* = 430 Female (68,7%)
	*N* = 75 Male (25%)	*N* = 182 Male (29,1%)
	*N* = 7 Diverse (2,3%)	*N* = 14 Diverse (2,2%)
Age	*M* = 25,2 (SD = 7,7 years)	*M* = 29,3 (SD = 10,1 years)
Occupation	*N* = 161 in employment (53,6%)	*N* = 413 in employment (66,0%)
	*N* = 173 student (57,7%)	*N* = 359 student (57,3%)
	*N* = 7 unemployed (2,3%)	*N* = 18 unemployed (18%)
	*N* = 2 retired (0,7%)	*N* = 14 retired (2,2%)
Confession	*N* = 133 Catholic (44,3%)	*N* = 225 Catholic (35,9%)
	*N* = 25 Protestant (8,3%)	*N* = 68 Protestant (10,9%)
	*N* = 10 other Christian religious communities (3,3%)	*N* = 22 other Christian religious communities (3,5%)
	*N* = 7 Islamic (2,3%)	*N* = 20 Islamic (3,2%)
	*N* = 1 Buddhist (0,3%)	*N* = 1 Judaist (0,2%)
	*N* = 56 no religion (18,7%)	*N* = 3 Buddhist (0,5%)
	*N* = 66 left their religion (22%)	*N* = 126 no religion (20,1%)
Nationality	*N* = 2 Other (0,7%)	*N* = 153 left their religion (24,4%) *N* = 8 Other (1,3%)
	*N* = 274 Austria, Germany or Swiss (91,3%)	*N* = 581 Austria, Germany or Swiss (92,8%)
	*N* = 18 EU (6,0%)	*N* = 33 EU (5,3%)
	*N* = 8 Non-EU (2,7%)	*N* = 12 Non-EU (1,9%)

### Principal component analysis of the OCEANic model structure

Principal component analysis was carried out using a VARIMAX rotation in order to determine the dimensional structure of the model. Based on the screeplot and theoretical consideration; in a next step the analysis was *a priori* set to two factors. Due to the non-normality of all both subscales, logarithmic transformations were performed (Kline, 2015).

Bartlett’s test [Chi–Square (231) = 2631,083, *p* < 0.001] and the Kaiser–Meyer–Olkin Measure of Sampling Adequacy (KMO = 0.887) indicated that the variables were suitable for factor analysis. Two factor solution explained 43.43% of the total variance. In the rotated factor solution, the first factor “OCEANic negative” was comprised of 13 items with factor loadings ranging from 0.76 to 0.37, showed an eigenvalue of 6.89 and explained 31.30% of the variance. The second factor “OCEANic positive” included nine items with eigenvalues of 2.67 (12.12%) and factor loadings ranging from 0.76 to 0.55. Moreover, the items comprising each factor, originally consisting of 13 items and 9 items, respectively, were reduced to six items through analysis of item-total correlation and careful consideration of construct validity.

### Confirmatory factor analysis of the OCEANic model

**FIGURE 1:**
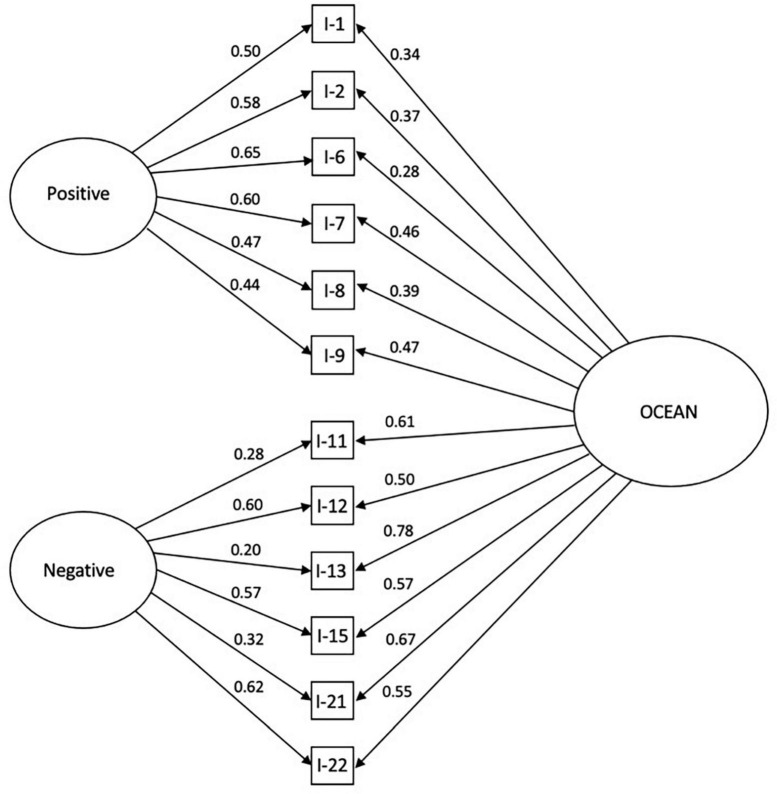
Bifactor model of the OCEANic scale.

**FIGURE 2:**
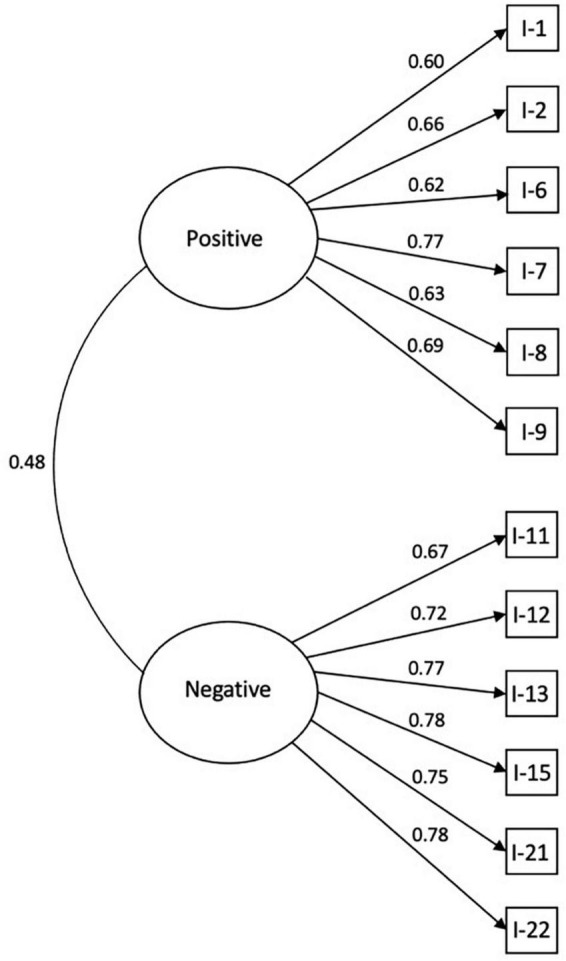
Two-factor model of the OCEANic scale.

Confirmatory factor analysis (CFA) was conducted testing a correlated model and a bifactorial model of the data. As seen in Table 2 both bifactor (RMSEA = 0.00) and correlated two-factor (RMSEA = 0.04) models exhibited excellent fit. See also Figures 1, 2 for further illustration.

**TABLE 2:** Global fit indices of both tested models.

Model	χ 2 (df)	RMSEA (90% CI)	CFI	NFI	TLI	SRMR
**12-Item-Version**						
Correlated two-factor model	107.148 (53)	0.040 (0.029−0.051)	0.99	0.98	0.99	0.048
Bifactor model	40.899 (42)	0.000 (0.000−0.028)	1.00	0.99	1.00	0.030

### Internal consistencies and correlations

Both subscales showed satisfactory internal consistency (OCEANic positive: α = 0.82; OCEANic negative: α = 0.88; *N* = 626). Spearman’s correlations were performed by using mean scores as well as factor scores based on the bifactor model of the OCEANic scales and mean scores of all other scales. Consequently, the factor scores and mean scores of the OCEANic scales showed almost identical correlation patterns. Therefore, only the correlations between the factor scores and mean scores of the other scales will be reported (see Table 3). Regarding spirituality, the positive subscale and the overall OCEANic scale showed the highest correlation (*r* = 0.63; *r* = 0.30; both *p* < 0.001). None of the other primary emotions or Big Five factors exhibited a correlation with spirituality higher than *r* = 0.10, supporting our hypothesis that spirituality is representing a distinct construct.

**TABLE 3:** Correlation between factor scores of OCEANic scales and mean scores of BANPS-GL, Spirituality and BFI.

	OCEAN total	OCEAN pos.	OCEAN neg.	Spirituality
OCEAN total	1	0.658**	0.902**	0.297**
OCEAN pos.	0.658**	1	0.342**	0.634**
OCEAN neg.	0.902**	0.342**	1	0.067
Spirituality	0.297**	0.634**	0.067	1
Openness to Experience	0.159**	0.198**	0.105**	0.098*
Conscientiousness	-0.061	0.092*	-0.123**	0.074
Extraversion	-0.139**	0.023	-0.215**	0.053
Agreeableness	-0.069	0.039	-0.078	0.045
Neuroticism	0.279**	-0.038	0.397**	-0.049
PLAY	-0.046	−044	-0.085*	-0.016
SEEKING	0.067	0.110**	0.057	0.000
FEAR	0.356**	0.048	0.486**	-0.063
ANGER	0.164**	0.048	0.160**	-0.006
SADNESS	0.416**	0.040	0.527**	-0.069
LUST	-0.187**	-0.073	-0.210**	-0.008
CARE	0.036	0.042	0.045	0.060

N = 626.

*p < 0.01,

**p < 0.001.

Furthermore, the positive subscale showed significant correlations with Openness to experience (*r* = 0.20; *p* < 0.001), SEEKING (*r* = 0.11; *p* < 0.001) and with Conscientiousness (*r* = 0.092; *p* < 0.01).

For the negative subscale, small to moderate correlations could be observed for SADNESS (*r* = 0.53; *p* < 0.001), FEAR (*r* = 0.49; *p* < 0.001), Neuroticism (*r* = 0.40; *p* < 0.001), ANGER (*r* = 0.17; *p* < 0.001), and Openness to Experience (*r* = 0.11; *p* < 0.001).

Extraversion (*r* = −0.22; *p* < 0.001) and LUST (*r* = −0.21; *p* < 0.001) as well as Conscientiousness (*r* = −0.12; *p* < 0.001) and PLAY (*r* = 0.09; *p* < 0.01) showed negative correlations with the negative subscale.

The overall factor showed significant positive correlations with SADNESS (*r* = 0.42; *p* < 0.001), FEAR (*r* = 0.36; *p* < 0.001), Neuroticism (*r* = 0.28; *p* < 0.001), ANGER (*r* = 0.16; *p* < 0.001), and Openness to Experience (*r* = 0.16; *p* < 0.001). Furthermore, there is a significant negative correlation between the overall factor and LUST (*r* = −0.19; *p* < 0.001) and Extraversion (*r* = −0.14; *p* < 0.001).

## Discussion

This study aimed to be a building block toward developing a self-rate measurement for the operationalization of oceanic feelings. To date no standardized questionnaire exists to measure oceanic feelings, based on concepts of Affective Neuroscience (AN). Psychometric properties outlined in this paper indicate strong reliability, robust structural validity, and a nomological network that aligns with our hypotheses. With respect to reliability, both subscales (positive and negative) exhibited good internal consistencies (Bühner, 2011). In accordance with current literature, principal component analysis with 22 items resulted in two factors. Regarding test economy and possible subsequent incorporation of the questionnaire to the BANPS-GL (Fuchshuber et al., 2023), the items were reduced to a total of 12 in terms of item selectivity, homogeneity and internal consistency. CFA suggested a well-fitting bifactorial structure, with one general factor and two residual factors that showed good model fit (Kline, 2023). These two residual factors reflect both components of oceanic feelings that include positive and negative affects.

Indicating that oceanic feelings originating in the PAG and spirituality might be interconnected systems, the highest correlation could be observed between spirituality and the positive subscale as well as the overall OCEANic scale (see Ferguson et al., 2022 for further discussion). As both subscales represent specific characteristics of oceanic feelings, results of correlations will be interpreted for each subscale separately. The positive subscale is intended to take up the feeling of self-dissolution and of oneness in a positive way, highest correlations could be reported between Openness to experience and SEEKING. Because the SEEKING system - often described as “the brain reward system”- is involved in motivation of general appetitive behaviors, the association to unity and embracement is not surprising (Montag and Panksepp, 2017). Relatedness to Openness to experience might be explained by willingness for experiencing transcendental feelings (see Unterrainer et al., 2014 for further discussion). Original works (Davis and Montag, 2019), as well as our study, found a significant correlation between Openness to experience and SEEKING (Hiebler-Ragger et al., 2018).

The negative subscale is intended to represent negative aspects of oceanic feelings e.g., the feeling of drowning, psychotic fragmentation or of all-enveloping darkness (cf. Unterrainer and Lewis, 2014). Therefore, highest correlations could be observed between all negative primary emotion systems - SADNESS, FEAR, and ANGER along with Neuroticism and negative correlations with Openness to Experience, PLAY and with LUST. Despite conceptual forms of depression, the proposed negative OCEANic factor has a strong focus on feelings of loneliness, despair, and hellish states of mind, which are usually linked to predominantly psychotic and paranoid experiences (Klein, 1935; Meltzer, 2003). Future research will be necessary to further investigate this relationship, however, it seems plausible that particularly major depressions with psychotic features may be closely linked to the negative OCEANic component.

## Limitations and future perspectives

Our findings should be seen as preliminary and require replication in future work. While oceanic feelings are often regarded as controversial and are stigmatized of being only present in highly religious/spiritual people, not all questions might have been answered truthfully. Although efforts were made to achieve diversity and representativeness in our sample, it predominantly consisted of young, female, and catholic participants. As a result, the internal and external validity, as well as the generalizability of our findings, may be compromised. Future research should consider collecting data from older participants and individuals from diverse cultural backgrounds to enhance the breadth and applicability of the results.

It might also be worthwhile investigating psychiatric patient groups (e.g., schizophrenic patients) with experience in substance use, especially intake of ketamine and/or psychedelic drugs as they are reported to induce oceanic feelings (cf. Unterrainer et al., 2013).

Furthermore, our questionnaire could subsequently be applied in dynamic brain imaging and cognitive neuroscience. Validity of our results for different cultural and religious backgrounds should be evaluated. Future studies should consider additional aspects of religion and spirituality and examine whether there is a relationship between oceanic feelings and psychopathologies as presence of religious/spiritual content in psychotic persons is often reported (Menezes and Moreira-Almeida, 2010).

## Conclusion

Based on our results, the newly developed OCEANic scale in its bifactorial form with two subscales and one general factor demonstrated overall convincing psychometric properties – high internal reliability, satisfying structural validity and plausible correlations with distinct facets of BANPS-GL and BFI-44. Further research is needed to thoroughly assess this questionnaire, particularly in terms of its external validity and its suitability for use in clinical populations. Further implementation as an additional module to the BANPS-GL might be considered.

## Data availability statement

The raw data supporting the conclusions of this article will be made available by the authors, without undue reservation.

## Ethics statement

The studies involving humans were approved by the Ethics committee of the University of Graz. The studies were conducted in accordance with the local legislation and institutional requirements. The participants provided their written informed consent to participate in this study.

## Author contributions

BS: Writing – original draft. JF: Writing – original draft. DA: Writing – review & editing. TP: Writing – review & editing. LR: Writing – review & editing. AF: Writing – review & editing. AS: Writing – review & editing. H-FU: Writing – original draft.
