# Supplementary material for: Is there an affective neuroscience of spirituality? The development and validation of the OCEANic feelings scale
Source: Front Hum Neurosci. 2024 Jan 17;18:1329226. doi: 10.3389/fnhum.2024.1329226 (PMC10828027; doi:10.3389/fnhum.2024.1329226)
Supplement: Supplementary file 1 [file Data_Sheet_1.docx]

# **Appendix**

***Translated version of the original questionnaire: OCEANic scale***

| 1 | 2 | 3 | 4 | 5 |
| --- | --- | --- | --- | --- |
| Strongly Disagree | Disagree | Neither Agree or Disagree | Agree | Strongly Agree |

| 1. I had an experience in which my person seemed to merge into something greater. | 1 | 2 | 3 | 4 | 5 |
| --- | --- | --- | --- | --- | --- |
| 2. I have experienced things that have made me realize that nothing is ever truly dead. | 1 | 2 | 3 | 4 | 5 |
| 3. I have experienced things that were so beautiful that they cannot be put into words. | 1 | 2 | 3 | 4 | 5 |
| 4. I had an experience that left me deeply affected. | 1 | 2 | 3 | 4 | 5 |
| 5. I had an experience through which I became aware of the meaning of life. | 1 | 2 | 3 | 4 | 5 |
| 6. In the great outdoors, I feel the presence of a higher power. | 1 | 2 | 3 | 4 | 5 |
| 7. There are living beings and/or objects to which I feel a psychic connection. | 1 | 2 | 3 | 4 | 5 |
| 8. I have already experienced once to merge with a living being and/or object. | 1 | 2 | 3 | 4 | 5 |
| 9. I have already experienced that inexplicable connections suddenly became understandable to me. | 1 | 2 | 3 | 4 | 5 |
| 10. I have had experiences that could have come from a past life. | 1 | 2 | 3 | 4 | 5 |
| 11. I once had the feeling of freezing inside from loneliness. | 1 | 2 | 3 | 4 | 5 |
| 12. I have had the feeling of falling into a deep, black hole before. | 1 | 2 | 3 | 4 | 5 |
| 13. I had an experience that was so horrible that it cannot be put into words. | 1 | 2 | 3 | 4 | 5 |
| 14. I have been beside myself with anger before. | 1 | 2 | 3 | 4 | 5 |
| 15. I have had the feeling of falling apart inside before. | 1 | 2 | 3 | 4 | 5 |
| 16. I have had the feeling of bursting with inner rage before. | 1 | 2 | 3 | 4 | 5 |
| 17. I know the feeling of freezing with fear. | 1 | 2 | 3 | 4 | 5 |
| 18. I have felt like I was losing my mind with rage before. | 1 | 2 | 3 | 4 | 5 |
| 19. I know the feeling of hatred that eats me up inside. | 1 | 2 | 3 | 4 | 5 |
| 20. I have had an experience that I cannot forgive. | 1 | 2 | 3 | 4 | 5 |
| 21. I had an experience that felt like I was in hell. | 1 | 2 | 3 | 4 | 5 |
| 22. I know the feeling of infinite despair. | 1 | 2 | 3 | 4 | 5 |

***Final Items:***

| 1 | 2 | 3 | 4 | 5 |
| --- | --- | --- | --- | --- |
| Strongly Disagree | Disagree | Neither Agree or Disagree | Agree | Strongly Agree |

| 1. I had an experience in which my person seemed to merge into something greater. | 1 | 2 | 3 | 4 | 5 |
| --- | --- | --- | --- | --- | --- |
| 2. I have experienced things that have made me realize that nothing is ever truly dead. | 1 | 2 | 3 | 4 | 5 |
| 6. In the great outdoors, I feel the presence of a higher power. | 1 | 2 | 3 | 4 | 5 |
| 7. There are living beings and/or objects to which I feel a psychic connection. | 1 | 2 | 3 | 4 | 5 |
| 8. I have already experienced once to merge with a living being and/or object. | 1 | 2 | 3 | 4 | 5 |
| 9. I have already experienced that inexplicable connections suddenly became understandable to me. | 1 | 2 | 3 | 4 | 5 |
| 11. I once had the feeling of freezing inside from loneliness. | 1 | 2 | 3 | 4 | 5 |
| 12. I have had the feeling of falling into a deep, black hole before. | 1 | 2 | 3 | 4 | 5 |
| 13. I had an experience that was so horrible that it cannot be put into words. | 1 | 2 | 3 | 4 | 5 |
| 15. I have had the feeling of falling apart inside before. | 1 | 2 | 3 | 4 | 5 |
| 21. I had an experience that felt like I was in hell. | 1 | 2 | 3 | 4 | 5 |
| 22. I know the feeling of infinite despair. | 1 | 2 | 3 | 4 | 5 |

***Original questionnaire in German language: OCEANic scale***

| 1 | 2 | 3 | 4 | 5 |
| --- | --- | --- | --- | --- |
| Starke Ablehnung | Ablehnung | Weder Ablehnung noch Zustimmung | Zustimmung | Starke Zustimmung |

| 1. Ich habe eine Erfahrung gemacht, in der meine Person in etwas Größerem aufzugehen schien. | 1 | 2 | 3 | 4 | 5 |
| --- | --- | --- | --- | --- | --- |
| 2. Ich habe Dinge erfahren, durch die mir bewusst wurde, dass nichts je richtig tot ist. | 1 | 2 | 3 | 4 | 5 |
| 3. Ich habe Dinge erfahren, die so schön waren, dass sie sich nicht in Worte fassen lassen. | 1 | 2 | 3 | 4 | 5 |
| 4. Ich habe eine Erfahrung gemacht, die mich zutiefst betroffen zurückließ. | 1 | 2 | 3 | 4 | 5 |
| 5. Ich habe eine Erfahrung gemacht, durch die mir der Sinn des Lebens bewusst wurde. | 1 | 2 | 3 | 4 | 5 |
| 6. In der freien Natur spüre ich die Gegenwart einer höheren Macht. | 1 | 2 | 3 | 4 | 5 |
| 7. Es gibt Lebewesen und/oder Gegenstände, zu denen ich eine übersinnliche Verbindung verspüre. | 1 | 2 | 3 | 4 | 5 |
| 8. Ich habe schon einmal erfahren, mit einem Lebewesen und/oder Gegenstand zu verschmelzen. | 1 | 2 | 3 | 4 | 5 |
| 9. Ich habe schon einmal erlebt, dass mir unerklärbare Zusammenhänge plötzlich verstehbar wurden. | 1 | 2 | 3 | 4 | 5 |
| 10. Ich habe Erfahrungen gemacht, die aus einem früheren Leben stammen könnten. | 1 | 2 | 3 | 4 | 5 |
| 11. Ich hatte schon einmal das Gefühl vor Einsamkeit innerlich zu erfrieren. | 1 | 2 | 3 | 4 | 5 |
| 12. Ich hatte schon einmal das Gefühl in ein tiefes, schwarzes Loch zu fallen. | 1 | 2 | 3 | 4 | 5 |
| 13. Ich habe eine Erfahrung gemacht, die so schrecklich war, dass sie sich nicht in Worte fassen lässt. | 1 | 2 | 3 | 4 | 5 |
| 14. Ich war schon einmal vor Zorn völlig außer mir. | 1 | 2 | 3 | 4 | 5 |
| 15. Ich hatte schon einmal das Gefühl innerlich zu zerfallen. | 1 | 2 | 3 | 4 | 5 |
| 16. Ich hatte schon einmal das Gefühl vor innerer Wut zu zerspringen. | 1 | 2 | 3 | 4 | 5 |
| 17. Ich kenne das Gefühl vor Angst zu erstarren. | 1 | 2 | 3 | 4 | 5 |
| 18. Ich hatte schon einmal das Gefühl vor Wut den Verstand zu verlieren. | 1 | 2 | 3 | 4 | 5 |
| 19. Ich kenne das Gefühl von Hass, der mich innerlich auffrisst. | 1 | 2 | 3 | 4 | 5 |
| 20. Ich habe eine Erfahrung gemacht, die ich nicht verzeihen kann. | 1 | 2 | 3 | 4 | 5 |
| 21. Ich habe eine Erfahrung gemacht, die sich anfühlte, als wäre ich in der Hölle. | 1 | 2 | 3 | 4 | 5 |
| 22. Ich kenne das Gefühl einer unendlichen Verzweiflung. | 1 | 2 | 3 | 4 | 5 |
